# Red Blood Cell Peroxynitrite Causes Endothelial Dysfunction in Type 2 Diabetes Mellitus via Arginase

**DOI:** 10.3390/cells9071712

**Published:** 2020-07-16

**Authors:** Ali Mahdi, John Tengbom, Michael Alvarsson, Bernhard Wernly, Zhichao Zhou, John Pernow

**Affiliations:** 1 Division of Cardiology, Department of Medicine Solna, Karolinska Institutet, Karolinska University Hospital, 171 77 Stockholm, Sweden; john.tengbom@ki.se (J.T.); bernhard@wernly.net (B.W.); john.pernow@ki.se (J.P.); 2 Division of Endocrinology and Diabetology, Department of Molecular Medicine and Surgery, Karolinska Institutet, Karolinska University Hospital, 171 77 Stockholm, Sweden; Michael.alvarsson@ki.se; 3 Division of Cardiology, Clinic of Internal Medicine II, Department of Cardiology, Paracelsus Medical University of Salzburg, 5020 Salzburg, Austria

**Keywords:** arginase, endothelial dysfunction, type 2 diabetes, red blood cells

## Abstract

We recently showed that red blood cells (RBCs) from patients with type 2 diabetes mellitus (T2DM-RBCs) induce endothelial dysfunction through a mechanism involving arginase I and reactive oxygen species. Peroxynitrite is known to activate arginase in endothelial cells. Whether peroxynitrite regulates arginase activity in RBCs, and whether it is involved in the cross-talk between RBCs and the vasculature in T2DM, is unclear and elusive. The present study was designed to test the hypothesis that endothelial dysfunction induced by T2DM-RBCs is driven by peroxynitrite and upregulation of arginase. RBCs were isolated from patients with T2DM and healthy age matched controls. RBCs were co-incubated with aortae isolated from wild type rats for 18 h in the absence and presence of peroxynitrite scavenger FeTTPS. Evaluation of endothelial function in organ chambers by cumulative addition of acetylcholine as well as measurement of RBC and vessel arginase activity was performed. In another set of experiments, RBCs isolated from healthy subjects (Healthy RBCs) were incubated with the peroxynitrite donor SIN-1 with subsequent evaluation of endothelial function and arginase activity. T2DM-RBCs, but not Healthy RBCs, induced impairment in endothelial function, which was fully reversed by scavenging of RBC but not vascular peroxynitrite with FeTPPS. Arginase activity was up-regulated by the peroxynitrite donor SIN-1 in Healthy RBCs, an effect that was inhibited by FeTTPS. Healthy RBCs co-incubated with aortae in the presence of SIN-1 caused impairment of endothelial function, which was inhibited by FeTTPS or the arginase inhibitor ABH. T2DM-RBCs induced up-regulation of vascular arginase, an effect that was fully inhibited by FeTTPS. Collectively, our data indicate that RBCs impair endothelial function in T2DM via an effect that is driven by a peroxynitrite-mediated increase in arginase activity. This mechanism may be targeted in patients with T2DM for improvement in endothelial function.

## 1. Introduction

Endothelial dysfunction plays a pivotal role in the development of vascular complications and is one of the early signs of vascular complications in type 2 diabetes mellitus (T2DM). It is characterized by an imbalance between vasoconstrictors such as enhanced reactive oxygen species (ROS) and vasodilators such as decreased bioavailability of nitric oxide (NO) [1,2]. The underlying causes and pathophysiological changes of the development of endothelial dysfunction in T2DM are complex and not completely understood [2]. As a consequence, therapeutic interventions that specifically target vascular complications are lacking, and there is a clinical need for identification of new therapeutic targets for improvement of cardiovascular outcomes among patients with T2DM. 

We recently unveiled a previously unknown mechanism underlying cardiovascular dysfunction in T2DM involving red blood cells (RBCs) referred to as *erythropathy* [3,4,5]. We were able to demonstrate that RBCs from patients with T2DM induce endothelial dysfunction, both in arteries isolated from healthy rats and in arteries from non-diabetic patients undergoing coronary artery bypass grafting [3]. The mechanisms behind these observations involve the up-regulation of arginase I, not only in RBCs in patients with T2DM but also in arteries, and, more specifically, endothelial cells, exposed to RBCs from this patient group [3]. This cross talk between RBC and the vasculature is mediated by an increase in ROS derived from NADPH oxidase (NOXs) [3]. Arginase, a metalloenzyme expressed with both isoforms, arginase I and II, in endothelial cells, and only arginase I in RBCs, among other cell types, regulates NO bioavailability by reciprocally inhibiting its formation through substrate competition of L-arginine [6,7]. Arginase activity is increased in the vasculature of animal models of T2DM as well as in the plasma of patients with T2DM and microvascular complications [8,9]. Additionally, we and others have, in early clinical interventions, shown that arginase inhibition improves endothelial function in several patient populations with cardiovascular risk factors [10]. This includes patients with T2DM where arginase inhibition improves both macro- [11,12] and microvascular [13] endothelial function, which is independent of glycemic control [12]. Our data suggest that RBC-derived arginase may be an important mediator of endothelial dysfunction and a putative therapeutic target. However, mechanistic insights on how RBC-arginase is regulated are lacking. Previous studies have suggested that the reactive nitrogen species (RNS) peroxynitrite is associated with endothelial dysfunction of T2DM and is a potent activator of arginase in endothelial cells [14,15]. Whether peroxynitrite regulates arginase activity in RBCs and is involved in the RBC-induced endothelial dysfunction is unclear and elusive. 

In this study, we test the hypothesis that peroxynitrite drives arginase activity in RBCs, which in turn activates arginase in endothelial cells in T2DM. To investigate this, we performed experiments in a co-incubation system using RBCs isolated from patients with T2DM as well as healthy subjects for ex-vivo assessment of endothelial function in the presence of peroxynitrite donors and scavenger. We also pharmacologically stimulated peroxynitrite formation in RBCs from healthy subjects in the presence of arginase inhibition to investigate its interaction with arginase.

## 2. Materials and Methods

### 2.1. Study Subjects

Eighteen patients with T2DM were recruited from the Endocrinology department and the Cardiology department at Karolinska University Hospital, Stockholm. Twenty healthy subjects matched for age and sex, free from medication and without a medical history of cardiovascular disease were also recruited. T2DM was defined according to the World Health Organization. Exclusion criteria were: any cardiovascular event including stroke and acute coronary syndrome in the past 6 months, ongoing treatment with warfarin or new oral anticoagulants, or age < 20 or > 80 years. T2DM was excluded among the healthy participants by an oral glucose tolerance test. Following an overnight fasting period, whole blood was collected in heparinized tubes with subsequent isolation of RBCs by immediate centrifugation at +4 °C and 1000× *g* for 10 min followed by three washing cycles with Krebs–Henseleit (KH) buffer [3]. Subject characteristics are summarized in Table 1.

Participants were informed of the nature, purpose and possible risk involved in the study. Oral and written informed consent were obtained from all the participants prior to inclusion in the study. The study was conducted according to the declaration of Helsinki and approved by the regional ethics committee (2014/463-31/3).

### 2.2. Animals

Wild-type rats from Sprague-Dawley or Wistar at age 9–16 weeks (Charles-River, Sulzfeld, Germany) were anesthetized with pentobarbital (50 mg/kg i.p.) followed by thoracotomy and isolation of aortic segments. Animal care and all protocols were approved by the regional ethical committee (17708-2019) and conformed to the Guide for Care and Use of Laboratory Animals published by the US National Institutes of Health (NIH publication no. 85-23, revised 1996).

### 2.3. RBC-Tissue Co-Incubation and Determination of Vascular Reactivity

Washed RBCs from patients with T2DM (T2DM-RBCs) and healthy subjects (Healthy RBCs) were diluted to a hematocrit of ~45% with KH buffer and incubated with rat aortic rings in cell culture incubator at 37 °C with 5% CO_2_ for 18 h [3]. Following the incubation, vessels were thoroughly washed and mounted in the wire myographs (Danish Myo Technology A/S, Hinnerup, Denmark). Endothelium-dependent relaxations (EDR) were determined by application of cumulatively increasing concentrations (10^−9^–10^−5^ M) of acetylcholine (ACh) to pre-constricted vascular segments with 9, 11-dideoxy-9α, 11α-methanoepoxy PGF_2α_ (U46619). A previous study [3] demonstrated that endothelium-independent relaxations are unaffected by T2DM-RBCs. To investigate the role of RBC peroxynitrite, the peroxynitrite scavenger Fe(III)5, 10, 15, 20-tetrakis(4-sulfonatophenyl) porphyrinato chloride (FeTPPS, 100 µM) was added in the co-incubation system for 18 h. The EDR was then assessed after thorough washing of the aortic segments. We have previously shown that there is no carry-over effect of ROS-scavengers in the co-incubation system on vascular function [3]. To investigate the role of vascular peroxynitrite, FeTPPS was added in the myograph organ chamber after the 18 h co-incubation with RBCs. To further confirm the role of peroxynitrite in the development of endothelial dysfunction in T2DM, Healthy RBCs were incubated with the peroxynitrite donor 3-morpholinosydnonimine hydrochloride (SIN-1, 500 µM) in the absence and presence of the arginase inhibitor 2(S)-amino-6-boronohexanoic acid (ABH) 10 mM [7] for 18 h, with subsequent evaluation of EDR. Additionally, similar experiments were performed in the absence and presence of FeTPPS (100 µM) to further confirm the specific up-regulation of peroxynitrite induced by SIN-1 at 500 µM. Control aortae were incubated with KH buffer in the absence and presence of FeTPPS or SIN-1. ABH does not affect vascular reactivity during 18 h incubation with KH, nor with Healthy RBCs [3]. To study the hypothesis that RBC peroxynitrite affects vascular arginase, another set of vessel segments incubated with T2DM-RBCs + FeTPPS were stored at −80 °C for measurement of arginase activity. Higher concentrations of compounds were needed to achieve proper stimulation or scavenging in co-incubations of RBCs and aortae due to the high density of RBCs (~45%) in these experiments [3].

### 2.4. Human RBC Incubations with SIN-1 and FeTTPS

To test the hypothesis that peroxynitrite is an activator of RBC arginase, washed Healthy RBCs were resuspended in KH-buffer (10% hematocrit) in a total volume of 1 mL and incubated for 24 h in the absence and presence of SIN-1 (25 µM) and FeTPPS (10 µM). The concentrations used were based on previous studies [15,16]. Following incubation, the mixture was centrifuged at +4 °C 1000× *g* for 10 min and the pellet was stored for arginase activity measurement. 

### 2.5. Tissue Extraction and Arginase Activity Assay

Human RBCs or rat aortas were lysed using RIPA lysis buffer (Amresco, Solon, OH, USA) containing protease inhibitors (Roche, Mannheim, Germany). Arginase activity was determined by a colorimetric assay as previously described. Briefly, 75 μL of 10 mM MnCl_2_ (mixed with TRIS 50 mM) with pH 7.5 was added to the samples (50 μL per sample). Arginase was activated by heating the mixture to 56 °C for 10 min. Hydrolysis of arginine was achieved by adding 50 μL of L-arginine (0.5 M dissolved in 50 mM TRIS, pH 9.7), and then incubated in 37 °C for 30 min (for RBCs) or 60 min (for aortas). The hydrolysis was stopped by adding 400 μL of stop-solution (H_2_SO_4_:H_3_PO_4_:H_2_O 1:3:7). After adding 25 μL of α-isonitrosopropiophenone (9% in ethanol), the mixture was incubated at 100 °C for 60 min. The mixture was then loaded onto a centrifugal filter and centrifuged for 5 min at 5000× *g* at room temperature. The concentration of the urea in filtrate was determined in a spectrophotometer (Wallac 1420 VICTOR2^TM^) at 540 nm. Arginase activity was calculated as urea production (mmol/mg protein/min) and expressed in percent of control.

### 2.6. Statistical Analyses

Baseline characteristics were analyzed with an unpaired Students *t*-test or Mann–Whitney test and expressed as means ± standard deviations. Normality was tested with pearson D’agostino test. EDR is presented as percent relaxation from preconstriction induced by U46619. And data are expressed as means ± standard error of the mean. Statistical analyses of EDR were performed with two-way analysis of variance (ANOVA), followed by Tukey’s post hoc test for multiple comparisons. Analysisof arginase activity data were performed with one-way ANOVA, followed by Tukey’s post hoc test for multiple comparisons. All statistical analyses were performed using Prism 7.0, GraphPad, San Diego, CA, USA. *p* < 0.05 was considered as statistically significant.

## 3. Results

### 3.1. Subjects

Baseline characteristics of the study subjects are presented in Table 1. Fasting blood glucose and glycated hemoglobin were markedly higher in patients with T2DM compared to healthy controls. Moreover, patients with T2DM had higher BMI and triglycerides but lower cholesterol levels compared to the healthy control group (Table 1). 

### 3.2. T2DM-RBCs Induce Endothelial Dysfunction through Peroxynitrite-Induced Arginase Activation in RBCs

**Figure 1:**
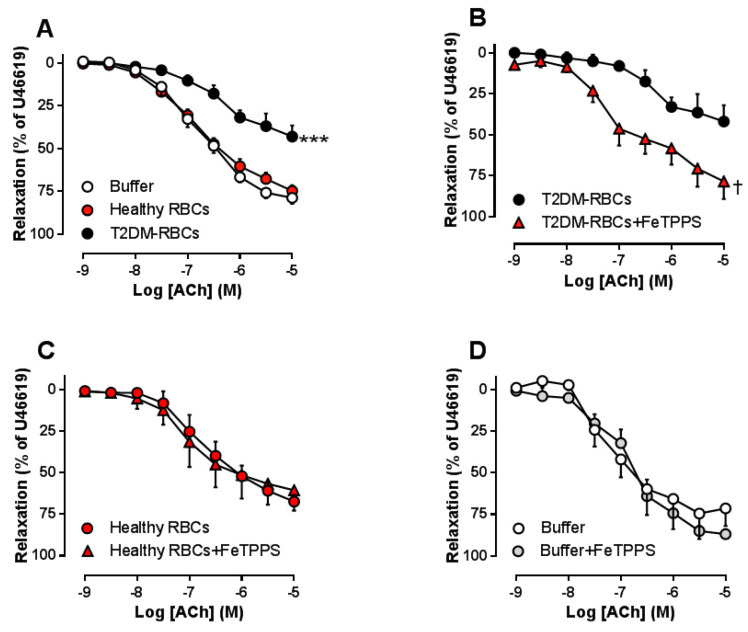
Effect of red blood cells from type 2 diabetes patients (T2DM-RBCs), healthy subjects (Healthy Red Blood Cells (RBCs)) or only buffer on endothelium-dependent relaxation (EDR) in rat aortas induced by acetylcholine (ACh) ((**A**), *n* = 9–16). The peroxynitrite scaveger FeTPPS was co-incubated with T2DM-RBCs ((**B**), *n* = 6), Healthy RBCs ((**C**), *n* = 3) and buffer ((**D**), *n* = 3). Values are mean ± SEM. *** *p* < 0.001 vs. Healthy RBCs or Buffer; ^†^ *p* < 0.05 vs. T2DM-RBCs.

In accordance with our previous studies [3,17], EDR was impaired in aortic segments incubated with T2DM-RBCs compared to those incubated with Healthy RBCs (Figure 1A). To test the hypothesis that RBC peroxynitrite is involved in this cross talk, the peroxynitrite scavenger FeTPPS was added in the RBC co-incubation system. FeTPPS completely prevented the development of endothelial dysfunction induced by T2DM-RBCs (Figure 1B), while it did not affect those aortic segments incubated with Healthy RBCs (Figure 1C) or buffer (Figure 1D). These observations indicate that RBC peroxynitrite is involved in endothelial dysfunction induced by T2DM-RBCs.

**Figure 2:**
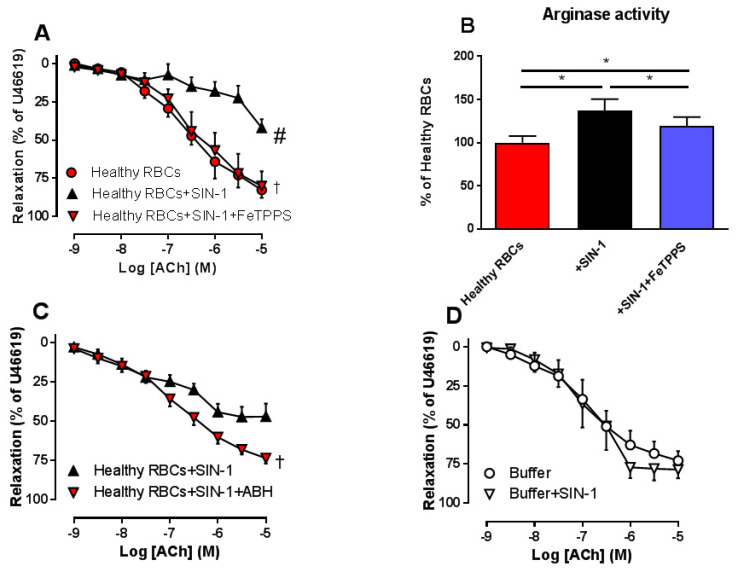
Effect of co-incubation of peroxynitrite donor SIN-1 with Healthy RBCs on EDR in rat aorta ((**A**), *n* = 5) and RBC arginase activity ((**B**), *n* = 8) in the absence and presence of FeTPPS, and on EDR in the absence and presence of the arginase inhibitor 2(S)-amino-6-boronohexanoic acid (ABH) ((**C**), *n* = 7). Effect of co-incubation of 3-morpholinosydnonimine hydrochloride (SIN-1) with buffer only on EDR ((**D**), *n* = 5–6). Values are mean ± SEM. ^#^ *p* < 0.05 vs. Healthy RBCs ^†^ *p* < 0.05 vs. Healthy RBCs+SIN-1; * *p* < 0.05 between groups in B.

Next, we investigated whether an increase in RBC peroxynitrite induces endothelial dysfunction. Administration of SIN-1 in the co-incubation of aortic segments with Healthy RBCs resulted in impairment of EDR, an effect that was fully reversed by FeTPPS (Figure 2A). This indicates that SIN-1 induces up-regulation of peroxynitrite, resulting in endothelial dysfunction. Since peroxynitrite is known to be a key trigger of arginase in endothelial cells in T2DM [15] and RBC arginase I is an important mediator causing endothelial dysfunction in T2DM, we hypothesized that peroxynitrite triggers arginase activity in RBCs, leading to endothelial dysfunction. Indeed, the peroxynitrite donor SIN-1 increased arginase activity in Healthy RBCs (Figure 2B). This increase was significantly attenuated by FeTPPS (Figure 2B). Furthermore, co-incubation of Healthy-RBCs with SIN-1, in the presence of the arginase inhibitor, reversed the impairment in EDR induced by SIN-1 (Figure 2C). To rule out the possibility that SIN-1 per se induces endothelial dysfunction, SIN-1 was incubated with aortic rings in the absence of RBCs, which did not affect EDR (Figure 2D), indicating that the peroxynitrite effect on EDR is mediated via the RBCs.

### 3.3. Involvement of Vascular Peroxynitrite in T2DM-RBC Induced Endothelial Dysfunction

**Figure 3:**
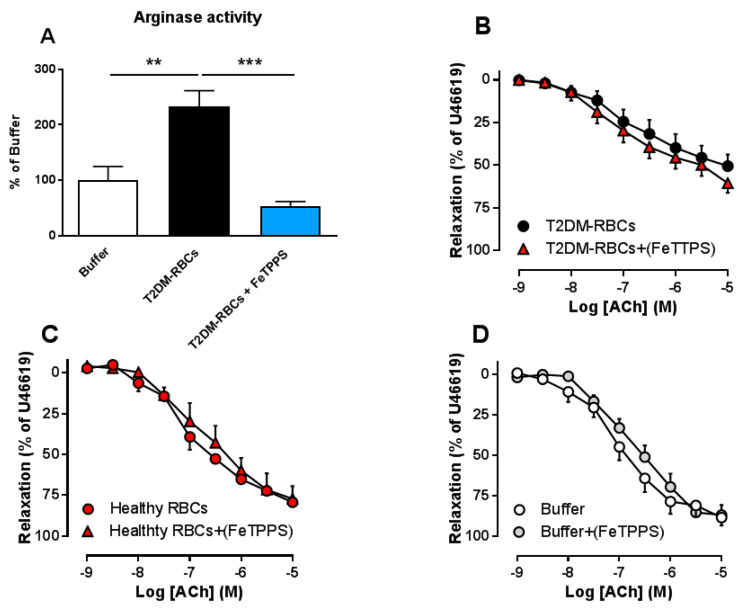
Arginase activity in aortic rings incubated with buffer or T2DM-RBCs in the absence and presence of FeTPPS ((**A**), *n* = 4–5). Effect of vascular scavenging of peroxynitrite with FeTPPS on EDR in rat aortas following incubation with T2DM-RBCs ((**B**), *n* = 6–7), Healthy RBCs ((**C**), *n* = 3) and Buffer ((**D**), *n* = 5). Values are mean ± SEM. Parentheses indicate that the scavenger was added in the organ baths following the 18 h incubation. Values are mean ± SEM. ** *p* < 0.01, *** *p* < 0.001 between groups in A.

As we previously showed that T2DM-RBCs induce a ROS-dependent increase in arginase activity in aortic segments [3], we hypothesized that one of the main species in this cross talk is peroxynitrite. In line with our previous results [3], vascular arginase activity was significantly elevated following incubation with T2DM-RBCs. This increase was completely inhibited by FeTPPS (Figure 3A). We previously confirmed that neither Healthy RBCs nor scavenging of ROS in Healthy RBCs affect vascular arginase, indicating that this effect is specific for T2DM-RBCs [3]. The impairment in EDR induced by T2DM-RBC was not affected by the scavenging of vascular peroxynitrite with FeTPPS (Figure 3B), suggesting that peroxynitrite in the vascular wall does not seem to be involved in endothelial dysfunction induced by T2DM-RBCs. Furthermore, FeTPPS did not affect EDR in aortic segments following incubation with Healthy RBCs (Figure 3C) or buffer (Figure 3D). Collectively, peroxynitrite induces up-regulation of arginase in the RBCs, which further induces up-regulation vascular arginase. However, T2DM-RBCs does not induce up-regulation of peroxynitrite in the vasculature.

## 4. Discussion

**Figure 4:**
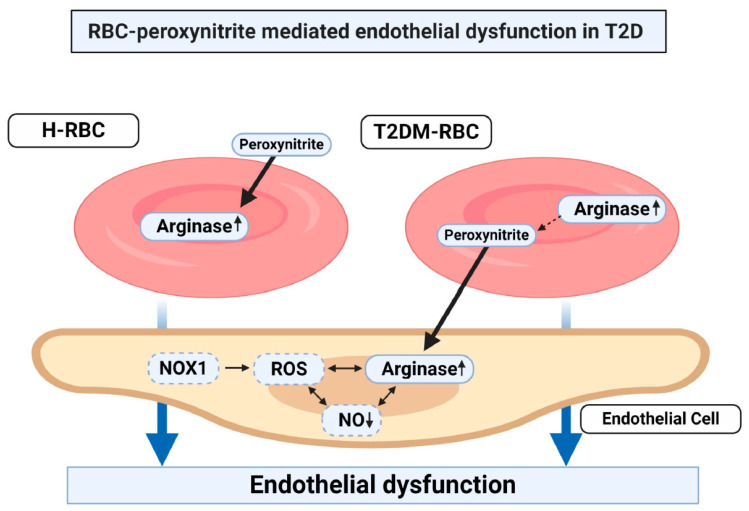
Central illustration. Peroxynitrite up-regulates arginase activity in red blood cells (RBCs) from healthy subjects (H-RBC) which induces endothelial dysfunction. Moreover, scavenging of peroxynitrite in RBCs from patients with T2DM (T2DM-RBCs) prevents endothelial dysfunction via prevention of up-regulation of arginase in endothelial cells. Dashed boxes represents earlier findings of ours where NADPH oxidase 1 (NOX1) represents a major source of reactive oxygen species (ROS), consequently resulting in the down-regulation of nitric oxide (NO) [3].

We previously observed a detrimental effect of T2DM-RBCs on endothelial function via a ROS-dependent increase in arginase I [3]. In the current study, we hypothesized that one of the major free radicals in this cross talk is peroxynitrite. Indeed, we were able observe that peroxynitrite scavenging in RBCs completely reversed the endothelial dysfunction induced by T2DM-RBCs (Figure 4). Moreover, our data demonstrate that up-regulation of arginase in T2DM-RBC and the vasculature following incubation with T2DM-RBCs is peroxynitrite-dependent (Figure 4). Lastly, we were able to induce erythropathy in RBCs from healthy subjects by stimulating peroxynitrite formation, which consequently not only up-regulated RBC arginase but also induced endothelial dysfunction (Figure 4). These data provide important mechanistic insights, demonstrating a crucial role of peroxynitrite as a mediator of RBC-induced endothelial dysfunction in T2DM.

Emerging evidence has revealed that RBCs contribute to vascular homeostasis and integrity in addition to their function as gas transporters [3,4,18,19]. Of further interest is that the function of RBCs is altered in several pathophysiological conditions [5,20]. In accordance with our previous findings [3,17], T2DM-RBCs induced endothelial dysfunction. One of the main mechanisms behind the development of dysfunction is through the ROS-mediated increase in arginase I, both in the vasculature and in the RBCs [3]. The human RBC is equipped with a well-developed antioxidant defense for maintenance of the redox balance [5]. However, this defense is compromised in various cardiovascular/metabolic diseases [5]. Supportive of this is the observation that ROS production is elevated in RBCs from patients with T2DM [3]. Interestingly, this increase is driven by arginase, and the increase in arginase activity is driven by ROS. Such mutual cross talk between ROS and arginase results in endothelial dysfunction [3]. In the current study, we demonstrate that peroxynitrite scavenging, specifically in RBCs, but not in the vasculature-attenuated endothelial dysfunction induced by T2DM-RBCs. Furthermore formation of peroxynitrite in Healthy RBCs impaired endothelial dysfunction, which identifies the key role of RBC peroxynitrite as mediator of RBC-induced endothelial dysfunction in T2DM.

A remaining question is which factors regulate arginase activity in RBCs. Previous data suggest that hyperglycemia might not be the sole trigger of arginase activity. We observed that incubation of RBCs with high glucose only marginally increased arginase activity [3]. Conversely, arginase activity was attenuated in T2DM-RBCs following optimization of glycemic control [17]. This suggests that glucose may be required for maintaining higher arginase activity in T2DM-RBCs rather than being a trigger of arginase activity. Here, we extend these previous findings and suggest that an alternative trigger of arginase in T2DM-RBCs is peroxynitrite. Accordingly, peroxynitrite has been described as activating arginase I expression in endothelial cells via Rho-kinase-dependent mechanisms [15]. The up-regulation of arginase in ischemia/reperfusion injury is also mediated via peroxynitrite [21]. Moreover, peroxynitrite scavenging improves diabetes-associated endothelial dysfunction [14]. In the present study, we have established a link between peroxynitrite and arginase by demonstrating that endothelial dysfunction induced by T2DM-RBC involves a peroxynitrite-mediated increase in arginase activity. Peroxynitrite was found to stimulate arginase activity in the RBCs. However, it remains unclear how peroxynitrite activates arginase and it is likely that multiple factors associated with T2DM drive this up-regulation of arginase.

Given the fact that RBCs do not contain a machinery for protein translation, up-regulation of the enzymatic activity of arginase may not be reflected in changes of arginase I protein expression, as previously observed in endothelial cells [15]. Hence, it is likely that other post-translational modifications of arginase occur, such as S-nitrosylation, which is the only described post-translational modification on arginase I [22]. Whether S-nitrosylation or other post-translational modifications on arginase regulating its activity, induced by peroxynitrite or other factors, occur in the RBC, is presently unclear. These modifications may include glycosylation or phosphorylation, which certainly deserve further attention.

In line with our previous findings [3], T2DM-RBCs increased vascular arginase activity. Of interest, peroxynitrite scavenging in T2DM-RBCs attenuated arginase activity in the vascular wall. However, peroxynitrite scavenging in the vascular wall failed to attenuate endothelial dysfunction induced by T2DM-RBC. Together with our previous demonstrations that T2DM-RBCs induced increased ROS production in the vasculature with subsequent arginase activation [3], these observations suggest that peroxynitrite in the T2DM-RBC is a major regulator of not only RBC but also vascular arginase. However, peroxynitrite does not seem to be one of the free radicals acting within the vasculature to induce endothelial dysfunction by T2DM-RBCs. Our data suggest that peroxynitrite derived from RBCs, in addition to activating arginase in the RBC, also induces arginase activity in the vascular endothelium, which in turn mediates the development of endothelial dysfunction. How this signaling is transmitted from the RBC to the vascular wall is unclear but it may be speculated that it occurs through extracellular carriers originating from the RBC. In a recent study, serum exosomes from T2DM patients contained large amounts of arginase I, which consequently triggered the impairment of endothelial function [23]. The source of these exosomes is unknown and it may be speculated that they originate from RBCs, as the RBC is a major source of extracellular microvesicles [24].

Some limitations of the current study deserve to be mentioned. As the experiments in this study were performed ex vivo, they may not fully reflect the in vivo situation. Furthermore, the patients in the current study had a rather poor glycemic control compared to a general diabetic population. However, we recently showed that endothelial dysfunction induced by T2DM-RBCs was not attenuated with RBCs from diabetic patients that had undergone intensive glycemic control [17], suggesting that the results in the current study may be generalized to a wider group of diabetes patients. Moreover, the majority of the subjects included in the current study were males, which makes it difficult to generalize the data to the general diabetic population.

In conclusion, the present study identifies the peroxynitrite-mediated increase in arginase as one of the triggers of erythropathy in T2DM. This mechanism may represent a future pharmacological target for improving vascular function among patients with T2DM.

## Figures and Tables

**Table 1:** Characteristics of healthy and T2DM subjects.

Variables:	Healthy *n* = 20	T2DM *n* = 18	
Age	60 ± 5	60 ± 13	^a^
No. of males	18	17	
BMI, kg/m^2^	26.3 ± 2.1	30.8 ± 4.4 ***	^a^
BP (mmHg):			
Systolic	135 ± 15	136 ± 20	^b^
Diastolic	82 ± 8	77 ± 10	^a^
Fasting glucose (mM)	5.6 ± 0.5	10.4 ± 3.6 ***	^a^
HbA1c, mmol/mol	35 ± 3	66 ± 23 ***	^b^
No. of smokers	0	2	
Hemoglobin, g/L	142 ± 9	141 ± 15	^a^
Creatinine, mmol/L	81 ± 10	85 ± 32	^b^
Triglycerides, mmol/L	1.1 ± 0.4	1.8 ± 1.0 **	^a^
Total cholesterol, mmol/L	5.1 ± 0.7	3.7 ± 1.2 ***	^b^
HDL, mmol/L	1.4 ± 0.3	1.0 ± 0.3 ***	^a^
LDL, mmol/L	3.2 ± 0.7	1.8 ± 0.9 ***	^b^
			
General medication, *n*			
ACEi/ARB	-	11	
Aspirin	-	10	
Lipid lowering	-	15	
b-blockers	-	4	
Calcium channel i	-	8	
Glucose lowering medication, *n* (%) dosage			
Insulin	-	12	
Metformin	-	10	
GLP1	-	6	
DPP-4i	-	3	
SU	-	5	
SGLT2i	-	3	

ACEi, angiotensin-converting enzyme; ARB, angiotensin II receptor blocker; BMI, body mass index; DPP-4i, dipeptidyl peptidase-4 inhibitor; GLP1, glucagon like peptide 1 analogue; HbA1c, glycated hemoglobin; HDL; high density lipoprotein; LDL, low density lipoprotein; SGLT2i, sodium-glucose cotransporter 2 inhibitor. ** *p* < 0.01, *** *p* < 0.001 vs. healthy. ^a^ Unpaired Students *t*-test, ^b^ Mann–Whitney test. Data are presented as means ± standard deviation.

## References

[1] Paneni F., Beckman J.A., Creager M.A., Cosentino F. (2013). Diabetes and vascular disease: Pathophysiology, clinical consequences, and medical therapy: Part I. Eur. Heart J..

[2] Gimbrone M.A., Garcia-Cardena G. (2016). Endothelial Cell Dysfunction and the Pathobiology of Atherosclerosis. Circ. Res..

[3] Zhou Z., Mahdi A., Tratsiakovich Y., Zahoran S., Kovamees O., Nordin F., Uribe Gonzalez A.E., Alvarsson M., Ostenson C.G., Andersson D.C. (2018). Erythrocytes From Patients With Type 2 Diabetes Induce Endothelial Dysfunction Via Arginase I. J. Am. Coll. Cardiol..

[4] Yang J., Zheng X., Mahdi A., Zhou Z., Tratsiakovich Y., Jiao T., Kiss A., Kovamees O., Alvarsson M., Catrina S.B. (2018). Red Blood Cells in Type 2 Diabetes Impair Cardiac Post-Ischemic Recovery Through an Arginase-Dependent Modulation of Nitric Oxide Synthase and Reactive Oxygen Species. JACC Basic Transl. Sci..

[5] Pernow J., Mahdi A., Yang J., Zhou Z. (2019). Red blood cell dysfunction: A new player in cardiovascular disease. Cardiovasc. Res..

[6] Pernow J., Jung C. (2016). The Emerging Role of Arginase in Endothelial Dysfunction in Diabetes. Curr. Vasc. Pharmacol..

[7] Yang J., Gonon A.T., Sjoquist P.O., Lundberg J.O., Pernow J. (2013). Arginase regulates red blood cell nitric oxide synthase and export of cardioprotective nitric oxide bioactivity. Proc. Natl. Acad. Sci. USA.

[8] Romero M.J., Platt D.H., Tawfik H.E., Labazi M., El-Remessy A.B., Bartoli M., Caldwell R.B., Caldwell R.W. (2008). Diabetes-induced coronary vascular dysfunction involves increased arginase activity. Circ. Res..

[9] Kovamees O., Shemyakin A., Pernow J. (2016). Amino acid metabolism reflecting arginase activity is increased in patients with type 2 diabetes and associated with endothelial dysfunction. Diabetes Vasc. Dis. Res..

[10] Mahdi A., Kovamees O., Pernow J. (2019). Improvement in endothelial function in cardiovascular disease—Is arginase the target?. Int. J. Cardiol..

[11] Shemyakin A., Kovamees O., Rafnsson A., Bohm F., Svenarud P., Settergren M., Jung C., Pernow J. (2012). Arginase inhibition improves endothelial function in patients with coronary artery disease and type 2 diabetes mellitus. Circulation.

[12] Mahdi A., Kovamees O., Checa A., Wheelock C.E., von Heijne M., Alvarsson M., Pernow J. (2018). Arginase inhibition improves endothelial function in patients with type 2 diabetes mellitus despite intensive glucose-lowering therapy. J. Intern. Med..

[13] Kovamees O., Shemyakin A., Checa A., Wheelock C.E., Lundberg J.O., Ostenson C.G., Pernow J. (2016). Arginase Inhibition Improves Microvascular Endothelial Function in Patients With Type 2 Diabetes Mellitus. J. Clin. Endocrinol. Metab..

[14] El-Remessy A.B., Tawfik H.E., Matragoon S., Pillai B., Caldwell R.B., Caldwell R.W. (2010). Peroxynitrite mediates diabetes-induced endothelial dysfunction: Possible role of Rho kinase activation. Exp. Diabetes Res..

[15] Chandra S., Romero M.J., Shatanawi A., Alkilany A.M., Caldwell R.B., Caldwell R.W. (2012). Oxidative species increase arginase activity in endothelial cells through the RhoA/Rho kinase pathway. Br. J. Pharmacol..

[16] Lauzier B., Sicard P., Bouchot O., Delemasure S., Moreau D., Vergely C., Rochette L. (2007). A peroxynitrite decomposition catalyst: FeTPPS confers cardioprotection during reperfusion after cardioplegic arrest in a working isolated rat heart model. Fundam. Clin. Pharmacol..

[17] Mahdi A., Jiao T., Yang J., Kövamees O., Alvarsson M., von Heijne M., Zhou Z., Pernow J. (2019). The Effect of Glycemic Control on Endothelial and Cardiac Dysfunction Induced by Red Blood Cells in Type 2 Diabetes. Front. Pharmacol..

[18] Cortese-Krott M.M., Rodriguez-Mateos A., Sansone R., Kuhnle G.G., Thasian-Sivarajah S., Krenz T., Horn P., Krisp C., Wolters D., Heiss C. (2012). Human red blood cells at work: Identification and visualization of erythrocytic eNOS activity in health and disease. Blood.

[19] Sun C.W., Yang J., Kleschyov A.L., Zhuge Z., Carlstrom M., Pernow J., Wajih N., Isbell T.S., Oh J.Y., Cabrales P. (2019). Hemoglobin beta93 Cysteine Is Not Required for Export of Nitric Oxide Bioactivity From the Red Blood Cell. Circulation.

[20] Zhou Z., Yang J., Pernow J. (2018). Erythrocytes and cardiovascular complications. Aging (Albany NY).

[21] Kiss A., Tratsiakovich Y., Gonon A.T., Fedotovskaya O., Lanner J.T., Andersson D.C., Yang J., Pernow J. (2014). The role of arginase and rho kinase in cardioprotection from remote ischemic perconditioning in non-diabetic and diabetic rat in vivo. PLoS ONE.

[22] Santhanam L., Lim H.K., Lim H.K., Miriel V., Brown T., Patel M., Balanson S., Ryoo S., Anderson M., Irani K. (2007). Inducible NO synthase dependent S-nitrosylation and activation of arginase1 contribute to age-related endothelial dysfunction. Circ. Res..

[23] Zhang H., Liu J., Qu D., Wang L., Wong C.M., Lau C.W., Huang Y., Wang Y.F., Huang H., Xia Y. (2018). Serum exosomes mediate delivery of arginase 1 as a novel mechanism for endothelial dysfunction in diabetes. Proc. Natl. Acad. Sci. USA.

[24] Kuo W.P., Tigges J.C., Toxavidis V., Ghiran I. (2017). Red Blood Cells: A Source of Extracellular Vesicles. Methods Mol./Biol./.

